# Association of genetic variants of Glutamate Metabotropic Receptor 5 gene and state-anhedonia

**DOI:** 10.1192/j.eurpsy.2022.398

**Published:** 2022-09-01

**Authors:** D. Török, X. Gonda, Z. Gal, N. Eszlari, G. Bagdy, G. Juhasz, P. Petschner

**Affiliations:** 1 Semmelweis University, Department Of Pharmacodynamics, Budapest, Hungary; 2 Semmelweis University, Department Of Psychiatry And Psychotherapy, Budapest, Hungary; 3 NAP-2-SE New Antidepressant Target Research Group, Hungarian Brain Research Program, Semmelweis University, Budapest, Hungary; 4 SE-NAP-2 Genetic Brain Imaging Migraine Research Group, Semmelweis University, Budapest, Hungary; 5 Institute of Chemical Research Kyoto University, Bioinformatics Center, Uji, Japan

**Keywords:** Glutamate, GRM5, Genetics, anhedonia

## Abstract

**Introduction:**

Andedonia is one of the core symptoms of depression. It is known that in case of depressed individuals experiencing anhedonia, the classical antidepressants are often ineffective, thus investigation of this symptom would be essential. Recent studies highlight the possible role of the glutamatergic system in anhedonia however, the genetic background of these assumptions is still unclear.

**Objectives:**

Our goal was to investigate the possible associations between state-anhedonia and genetic variants from *GRM5* (Glutamate Metabotropic Receptor 5) gene.

**Methods:**

For our analysis we used data from the NewMood (New Molecules in Mood Disorders, LSHM-CT-2004-503474) project. Participants (n = 1820) aged between 18-60, were recruited in Budapest and in Manchester. All volunteers filled out mental-health questionnaires and provided DNA sample. Genotyping was performed by Illumina’s CoreExom PsychChip. Altogether 1282 variants from *GRM5* gene survived the genetic quality control steps. State-anhedonia was measured with an item from the Brief Symptom Inventory questionnaire. We performed logistic regression using Plink 2.0. During our analyses, age, gender, population and the top10 principal components of the genome were added into the model as covariates. Correction for linkage-disequilibrium were performed with LDlink.

**Results:**

After the correction of linkage-disequilibrium, three independent variables (r^2^<0.2), (rs1827603, rs6483520, rs35669869) yielded significant (p<0.05) results, both in additive and in dominant model. In case of recessive model, only rs11020880 showed significant (p<0.05) effect.

**Conclusions:**

The detected nominally significant associations between state-anhedonia and genetic variants from *GRM5* gene strengthen previous assumptions about the possible relationship between glutamatergic system and anhedonia.

**Disclosure:**

This study was supported by: MTA-SE Neuropsychopharmacology and Neurochemistry Research Group; 2017-1.2.1-NKP-2017-00002; KTIA_13_NAPA-II/14; KTIA_NAP_13-1-2013- 0001; KTIA_NAP_13-2- 2015-0001; 2020-4.1.1.-TKP2020 and 2019-2.1.7-ERA-NET-2020-00005 (TRAJEC

